# Nucleophosmin/B23 is a negative regulator of estrogen receptor α expression via AP2γ in endometrial cancer cells

**DOI:** 10.18632/oncotarget.11048

**Published:** 2016-08-04

**Authors:** Chiao-Yun Lin, Angel Chao, Tzu-Hao Wang, Li-Yu Lee, Lan-Yan Yang, Chia-Lung Tsai, Hsin-Shih Wang, Chyong-Huey Lai

**Affiliations:** ^1^ Department of Obstetrics and Gynecology, Chang Gung Memorial Hospital and Chang Gung University, College of Medicine, Taoyuan, Taiwan; ^2^ Gynecologic Cancer Research Center, Chang Gung Memorial Hospital, Taoyuan, Taiwan; ^3^ Genomic Medicine Research Core Laboratory, Chang Gung Memorial Hospital, Taiwan; ^4^ Department of Clinical Pathology, Chang Gung Memorial Hospital and Chang Gung University, College of Medicine, Taoyuan, Taiwan; ^5^ Statistics, Clinical Trial Center, Chang Gung Memorial Hospital, Taipei City, Taiwan; ^6^ Graduate Institute of Clinical Medical Sciences, Chang Gung University, Taoyuan, Taiwan

**Keywords:** endometrial cancer, nucleophosmin, estrogen receptor α, activator protein-2γ, hormonal therapy

## Abstract

Endometrial cancers expressing estrogen and progesterone receptors respond to hormonal therapy. The disappearance of steroid hormone receptor expression is common in patients with recurrent disease, ultimately hampering the clinical utility of hormonal therapy. Here, we demonstrate for the first time that nucleophosmin (NPM1/B23) suppression can restore the expression of estrogen receptor α (ESR1/ERα) in endometrial cancer cells. Mechanistically, B23 and activator protein-2γ (TFAP2C/AP2γ) form a complex that acts as a transcriptional repressor of ERα. Our results indicate that B23 or AP2γ knockdown can restore ERα levels and activate ERα-regulated genes (e.g., cathepsin D, EBAG9, and TFF1/pS2). Moreover, AP2γ knockdown in a xenograft model sensitizes endometrial cancer cells to megesterol acetate through the upregulation of ERα expression. An increased immunohistochemical expression of AP2γ is an adverse prognostic factor in endometrial cancer. In summary, B23 and AP2γ may act in combination to suppress ERα expression in endometrial cancer cells. The inhibition of B23 or AP2γ can restore ERα expression and can serve as a potential strategy for sensitizing hormone-refractory endometrial cancers to endocrine therapy.

## INTRODUCTION

Endometrial cancer is the most common cancer of the female reproductive tract in industrialized countries [1]. In Taiwan, the incidence of endometrial cancer has increased 10-fold over the last 30 years (from <100 cases per year to >1000 cases annually), and currently ranks first among gynecologic malignancies [2]. Major prognostic factors include histological type, histological differentiation, disease stage, myometrial invasion, lymph node metastases, and adnexal metastases. Well-differentiated tumors generally express estrogen and progesterone receptors [3–5] and respond to hormonal therapy [6, 7]. However, the disappearance of steroid hormone receptor expression is common in patients with recurrent estrogen-related cancers, ultimately hampering the clinical utility of hormonal therapy [8, 9].

The expression of estrogen receptor 1 (ESR1/ERα, gene ID 2099) is tightly regulated in a coordinated fashion by numerous transcription factors, including retinoic acid receptor-α (RARα), PAX2, GATA3, NKX3.1, LEF1, FOXA1, and activator protein-2γ (TFAP2C/AP2γ, gene ID 7022) [10–16]. The AP2 family of transcription factors consists of five different members (termed α, β, γ, δ, and ε) [17]. AP2 proteins consist of homo - and heterodimers that bind to GC-rich DNA sequences and are able to either activate or repress target genes [18]. AP2 can contribute to tumorigenesis by regulating the expression of specific target genes involved in growth control, including ER [19], HER2 [20], and CDKN1A [21].

Strategies that promote re-expression of hormonal receptors may allow patients with recurrent endometrial cancer to resume endocrine treatment. Epigenetic silencing of estrogen receptors and progesterone receptors is common in endometrial cancer cells [22, 23]. Consequently, epigenetic modulation has recently emerged as a novel therapeutic target for overcoming hormonal therapy resistance. Combined treatment with the DNA methyltransferase (DNMT) inhibitor 5-aza-2′-deoxycytidine (5-aza-dC) and the histone deacetylase (HDAC) inhibitor trichostatin A stimulates the expression of ERα in ER-negative breast cancer cells [24]. Interestingly, the reactivation of ERα can synergistically be enhanced by the concomitant dietary use of genistein and trichostatin A [25]. Recently, Ning et al. [22] demonstrated that IL17A secreted from macrophages can induce ERα expression through TET1-mediated epigenetic modulation. Similarly, HDAC inhibitors may restore progesterone receptor expression [23].

Nucleophosmin (NPM1/B23, gene ID 4869) is a nucleolar phosphoprotein implicated in ribosome biogenesis, centrosome duplication, cell cycle progression, apoptosis, and cell differentiation [26]. There is a growing interest in the role of B23 in human solid malignancies, including endometrial [27], ovarian [28], thyroid [29], gastric [30], colon [31], prostate [32], and bladder [33, 34] cancer. B23 is highly expressed in proliferating cells [35] and has been implicated in the pathogenesis of endometrial cancer [27]. We have previously shown that estrogens activate B23 via ERα receptors [27]. In the current study, we demonstrate the existence of a negative feedback loop through which an increased B23 expression suppresses ERα. Because suppression of ERα expression is a late event during estrogen-dependent endometrial tumorigenesis, the inhibition of B23 may represent a strategy to promote ERα re-expression that ultimately restores tumor sensitivity to hormonal therapy.

## RESULTS

### ERα expression is reduced in high-passage RL95-2 and Ishikawa endometrial cancer cells

**Figure 1:**
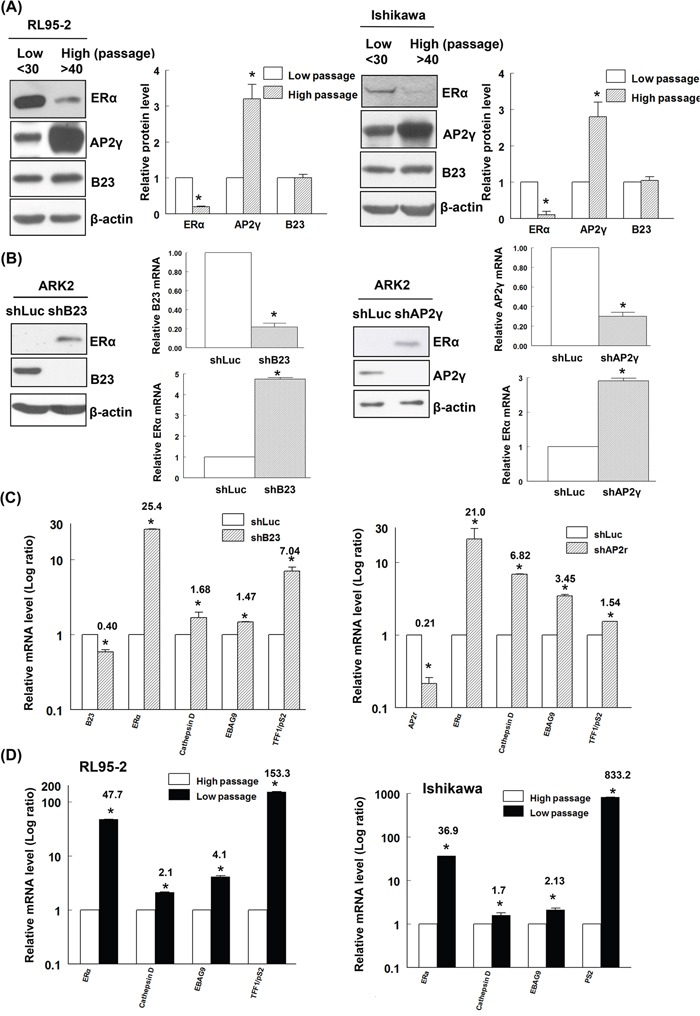
B23 negatively regulates ERα expression and its downstream genes in endometrial cancer cells **A.** Early-passage (n <30) and late-passage (n >40) RL95-2 and Ishikawa endometrial cancer cells were subcultured in medium containing phenol red every 2−3 days. Equal amounts of protein lysates were separated by SDS-PAGE and subjected to immunoblotting with antibodies raised against ERα, AP2γ, B23, and β-actin. The presence of an equal amount of proteins in each lane was confirmed with β-actin. In the quantitative bar graph, the results are expressed as means ± standard errors of the mean. Data are from three independent experiments. *P <0.05 compared with controls. **B.** ERα-negative ARK2 cells were transiently transfected with shLuc (control), shB23 (left panel), or shAP2γ (right panel) for 72 h and immunoblotted with the reported antibodies. The presence of an equal amount of proteins in each lane was confirmed with β-actin. RNAs were analyzed with real-time qPCR using the reported primers. In the quantitative bar graph, the results are expressed as means ± standard errors of the mean. **C.** ARK2 cells were transiently transfected with shLuc or shB23 or shAP2γ for 72 h. The resulting RNAs of B23, AP2γ, ERα, cathepsin D, EBAG9, and TFF1/pS2 were analyzed with real-time qPCR using the reported primers. **D.** RL95-2 and Ishikawa cells of different passages were collected and the resulting RNAs of ERα, cathepsin D, EBAG9, and TFF1/pS2 were analyzed with real-time qPCR using the reported primers. Data are expressed as means ± standard errors from three independent experiments. * *P* <0.05 compared with controls.

We initially examined ERα and PR expression in both low-passage (passages < 30) and high-passage (passages > 40) RL95-2 and Ishikawa endometrial cancer cell lines. High-passage RL95-2 and Ishikawa cells showed a reduced ERα expression (Figure 1A) whereas PR expression was not significantly altered (Supplementary Figure S1a). Because the ERα promoter region is characterized by the presence of an AP2γ binding site [16, 36], we quantified the cellular concentrations of AP2γ protein. Increased AP2γ expression levels were evident in high-passage RL95-2 and Ishikawa cells (Figure 1A).

### B23 negatively regulates ERα expression and its downstream genes through AP2γ

Owing to their ease of transfection, ERα-negative ARK2 endometrial cancer cells were used for transfection experiments. Surprisingly, knockdown of B23 or AP2γ was found to restore ERα expression (both at the protein and mRNA levels) in ARK2 cells (Figure 1B). However, no effects on PR expression were evident in ARK2, RL95-2 and Ishikawa endometrial cancer cells (Supplementary Figures S1b and S1c). Consequently, all other experiments were focused on ERα. Knockdown of B23 or AP2γ activated distinct ERα-regulated genes, including cathepsin D, EBAG9, and TFF1/pS2 (Figure 1C). Because of the lack of estrogen receptors expression in high-passage ERα-negative endometrial cancer cells, ERα-activated genes were found to be downregulated (Figure 1D).

We also tested whether B23 can regulate the expression of ERα in different breast cancer cells. The reactivation of ERα through the inhibition of B23 or AP2γ was confirmed in ERα-negative MDA-MBA231 and Hs578T breast cancer cells (Supplementary Figure S2a). In contrast, inhibition of B23 and AP2γ decreased estrogen receptor expression in ERα-positive MCF and T47D cells (Supplementary Figure S2b). These results suggest that B23/AP2γ-mediated regulation of ERα expression differs according to the pre-existing presence or absence of the estrogen receptor. In line with this possibility, a previous report demonstrated that AP2γ was able to induce ERα promoter expression in hormone-responsive breast cancer [15].

### B23 interacts with AP2γ and suppresses ERα

**Figure 2:**
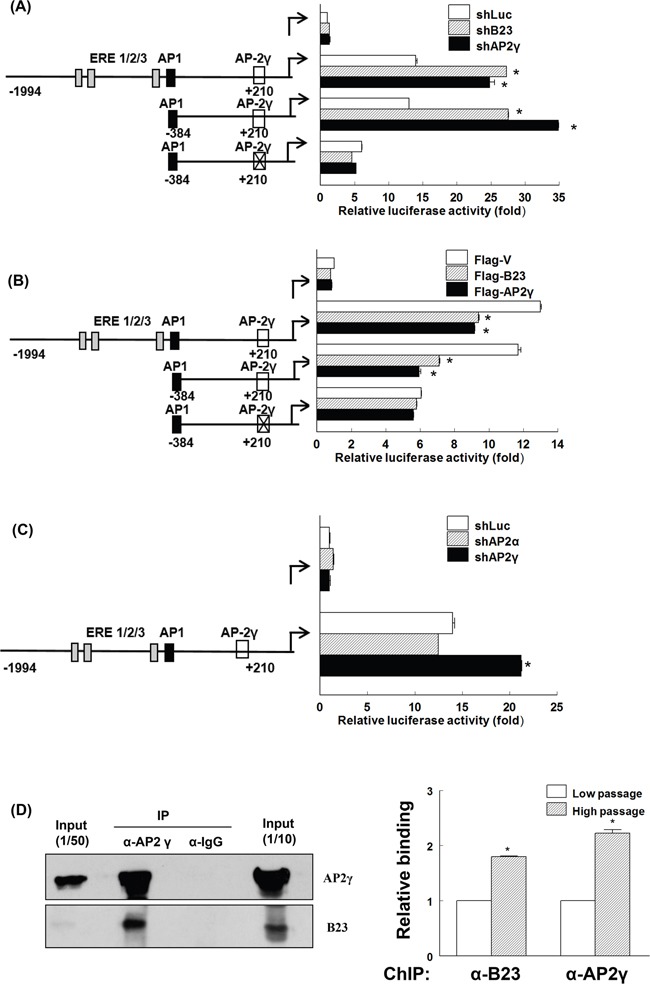
AP2γ is required for B23-mediated suppression of ERα promoter expression **A, B,** and **C.** ARK2 cells were transiently co-transfected with reporter constructs of different lengths or containing the mutated AP2γ binding site of the ERα promoter as well as shLuc, shB23, shAP2γ, or shAP2α (A, C); flag-V, flag-B23, or flag-AP2γ (B) for 48 h. Protein lysates were then assayed for luciferase and β-galactosidase activities. The relative promoter activity was normalized to that of β-galactosidase. The schematic diagram on the left depicts the location of different response elements in the promoter. X indicates the mutated AP2γ site. **D.** Whole-cell lysates were immunoprecipitated (IP) with anti-AP2γ and subsequently analyzed by immunoblotting with antibodies raised against AP2γ (left upper panel) and B23 (left lower panel). A control antibody (IgG) was used for mock immunoprecipitation. HC: heavy chain, LC: light chain. Chromatin immunoprecipitation (ChIP) assays (right panel) were performed using chromatin fragments prepared from Ishikawa cells. Cell lysates were prepared and immunoprecipitated with control (IgG), anti-B23, and anti-AP2γ antibodies. The immunoprecipitated genomic regions were assayed with real-time qPCR using primers that encompassed the AP2γ binding site in the ERα promoter.

We then generated luciferase reporter constructs of a truncated ERα promoter with the goal of identifying the domain mediating B23 suppression on the ERα promoter. The reporter activity of the construct ranging from position −1994 to +210 of the ERα promoter (containing ERE, AP1, and AP2γ recognition sequences) was significantly increased by B23 or AP2γ knockdown (Figure 2A). Mutations in the AP2γ binding site − but not deletions of ERE and AP-1 recognition sequences − abolished the stimulation of the ERα promoter reporter elicited by B23 or AP2γ knockdown (Figure 2A). The suppressive function of AP2γ was confirmed by the observation that B23 and AP2γ overexpression inhibited ERα promoter reporter activity (Figure 2B). Although AP2α forms a complex with B23 during retinoic-acid-induced cell differentiation [37], our results obtained in endometrial cancer cells do not support an involvement of AP2α in the regulation of ERα promoter (Figure 2C).

We also examined the effects of B23 or AP2γ knockdown on ERα promoter reporter activity and expression of ERα downstream genes during estrogen treatment. Estrogens induced a mild stimulation of ERα promoter activity in ARK2 cells, with a lower magnitude compared with that elicited by B23 or AP2γ knockdown (Supplementary Figure S3). Estrogen treatment also amplified the stimulation of ERα downstream genes caused by B23 or AP2γ knockdown (Supplementary Figure S4). Taken together, these results indicate that B23 or AP2γ knockdown stimulates the reporter activity of ERα promoter as well as ERα downstream genes has slight effect on estrogen treatment.

We subsequently examined whether B23 and AP2γ interact with each other. To this aim, the immunoprecipitation (IP) and chromatin immunoprecipitation (ChIP) assays were performed using antibodies raised against B23 and AP2γ. The results of IP and ChIP confirmed that B23 forms a complex with AP2γ at the AP2γ-binding site of the ERα promoter (Figure 2D and Supplementary Figure S5). Taken together, our findings indicate that AP2γ − a negative regulator of the ERα promoter − mediates the suppression of ERα transcription elicited by B23.

### B23 forms a protein complex with AP2γ that promotes its stabilization

**Figure 3:**
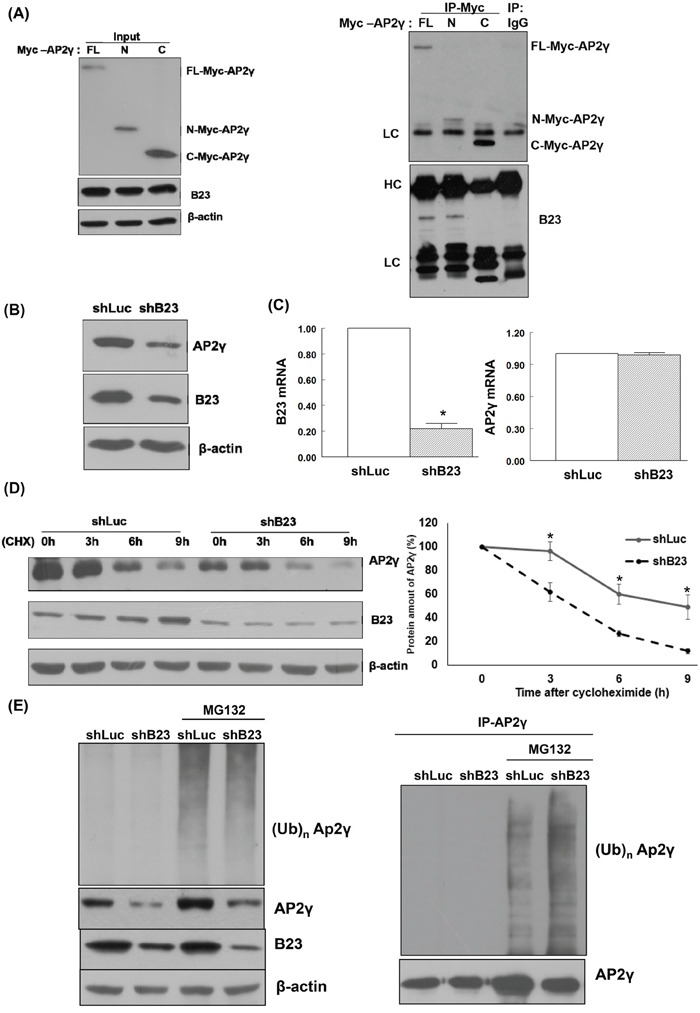
B23 interacts with the N-terminal fragment of AP2γ **A.** Lysates from ARK2 cells transiently expressing Myc-tagged AP2γ (FL, N, or C) were separated by SDS-PAGE and subjected to immunoblotting with antibodies raised against Myc, B23, and β-actin. The presence of an equal amount of proteins in each lane was confirmed with β-actin. Whole-cell lysates were immunoprecipitated (IP) with anti-Myc, and subsequently analyzed by immunoblotting with antibodies raised against Myc (upper panel) and B23 (lower panel). A control antibody (IgG) was used for mock immunoprecipitation. HC: heavy chain, LC: light chain. **B.** ARK2 cells were transiently transfected with shLuc or shB23 for 72 h, and subjected to immunoblotting with the reported antibodies (left panel). The presence of an equal amount of proteins in each lane was confirmed with β-actin. **C.** ARK2 cells were transiently transfected with shLuc or shB23 for 72 h and RNAs were analyzed with real-time qPCR using the reported primers. In the quantitative bar graph, the results are expressed as means ± standard errors of the mean. **D.** ARK2 cells were transiently transfected with shLuc or shB23 for 72 h and treated with 25 μg/mL cycloheximide (CHX). Cell lysates were prepared at the reported time points. Western blot analysis was performed using AP2γ, B23, and β-actin antibodies. The amount of AP2γ protein at each time point was quantified based on normalized AP2γ levels in ARK2 cells at 0 h (right panel). Data are expressed as means ± standard errors from three independent experiments. **E.** ARK2 cells were treated with shLuc or shB23 for 72 h and 10 μM MG132 for 24 h. Whole-cell lysates prepared in WCE lysis buffer were subjected to immunoblot for identifying the proteins of interest (left panel). The right panel shows the immunoprecipitation performed with the anti-AP2γ antibody probed with anti-ubiquitin (Ub, top) and AP2γ antibodies (bottom). A control antibody (IgG) was used for mock immunoprecipitation.

Full-length as well as N-terminal and C-terminal fragments of the AP2γ protein were generated using specific constructs. Endogenous B23 was able to interact with both the full-length form and the N-terminal fragment of AP2γ, but not with its C-terminal fragment (Figure 3A). Knockdown of B23 significantly decreased AP2γ protein levels (Figure 3B) but not AP2γ mRNA expression (Figure 3C). Cycloheximide (CHX) − a translational inhibitor that blocks the synthesis of new proteins − was used to investigate the role of B23 in the post-translational regulation of AP2γ. The results indicated that B23 was able to stabilize AP2γ protein. Accordingly, the degradation of AP2γ was accelerated when B23 was knocked down in ARK2 cells (Figure 3D). Treatment with MG132 − a proteasome inhibitor − increased AP2γ protein levels in B23-depleted cells (Figure 3E). An increased accumulation of AP2γ-ubiquitin conjugates in B23-depleted cells exposed to MG132 further confirmed that the AP2γ protein stability is governed by ubiquitin-mediated proteasome degradation.

### Restoration of ERα sensitizes endometrial cancer cells to megestrol acetate

**Figure 4:**
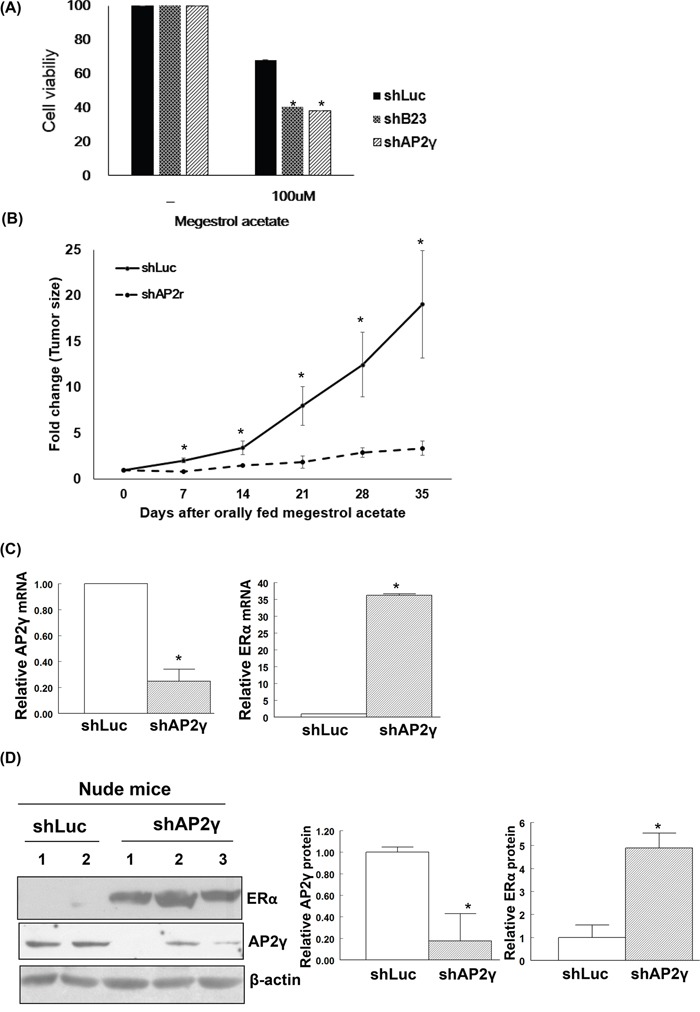
Restoration of ERα expression renders endometrial cancer cells susceptible to megestrol acetate treatment **A.** Human endometrial cancer Ishikawa/shLuc (shLuc), Ishikawa/shB23 (shB23), and Ishikawa/shAP2γ (shAP2γ) cells were treated with 100 nM megestrol acetate for 72 h and assayed by MTT. Nude mice experiments are shown in (B-D). **B.** Knock-down cell lines Ishikawa/shLuc (shLuc), Ishikawa/shB23 (shB23), and Ishikawa/shAP2γ (shAP2γ) were inoculated into nude mice; tumor volumes were subsequently measured on a weekly basis. No tumor formation was observed when animals were inoculated with the stable B23 knock-down cell line. Tumor-bearing nude mice orally received megestrol acetate (10 mg/kg) dissolved in cooking oil (5 days per week). The points depict the means (± standard deviations) of tumor volumes for megestrol acetate-treated (n = 3) and control animals (n = 2). *P < 0.05 compared with controls. After treatment with megestrol acetate, tumors derived from Ishikawa/shLuc (shLuc) and Ishikawa/shAP2γ (shAP2γ) cells were analyzed for mRNA (C) and protein levels (D). **C.** RNAs were analyzed with real-time qPCR with the reported primers. In the quantitative bar graph, the results are expressed as means ± standard errors of the mean. **D.** Cell lysates immunoblotted with ERα and AP2γ antibodies. The presence of an equal amount of proteins in each lane was confirmed with β-actin. In the quantitative bar graph, the results are expressed as mean ± standard error of the mean.

Knockdown of B23 or AP2γ in Ishikawa endometrial cancer cells abrogated cell viability and significantly increased their susceptibility to megestrol acetate (Figure 4A). To further examine the *in vivo* effects of B23 and AP2γ, Ishikawa/shLuc, Ishikawa/shB23, and Ishikawa/shAP2γ stable clones were subcutaneously injected in the flank of female nude mice. The injection of Ishikawa/shB23 cells did not result in tumor formation. Notably, tumor growth was significantly inhibited by treatment with megestrol acetate only in animals injected with Ishikawa/shAP2γ cells, but not with Ishikawa/shLuc (control) cells (P < 0.05; Figure 4B). Moreover, AP2γ knock-down restored the expression of ERα in Ishikawa cells at both mRNA (Figure 4C) and protein levels (Figure 4D). Similarly, NSC348884 − a B23 inhibitor − suppressed B23 and increased ERα expression in both ARK2 and Ishikawa cells (Supplementary Figures S6a and S6b). The tumor volume in NSC348884-treated mice was significantly lower than that observed in vehicle-treated mice (P < 0.05) (Supplementary Figures S6c and S6d). In addition, higher ERα expression levels were evident in NSC348884-treated tumor samples compared with vehicle-treated mice (Supplementary Figure S6e).

### Adverse prognostic significance of high AP2γ or low ERα expression in endometrial cancer

**Figure 5:**
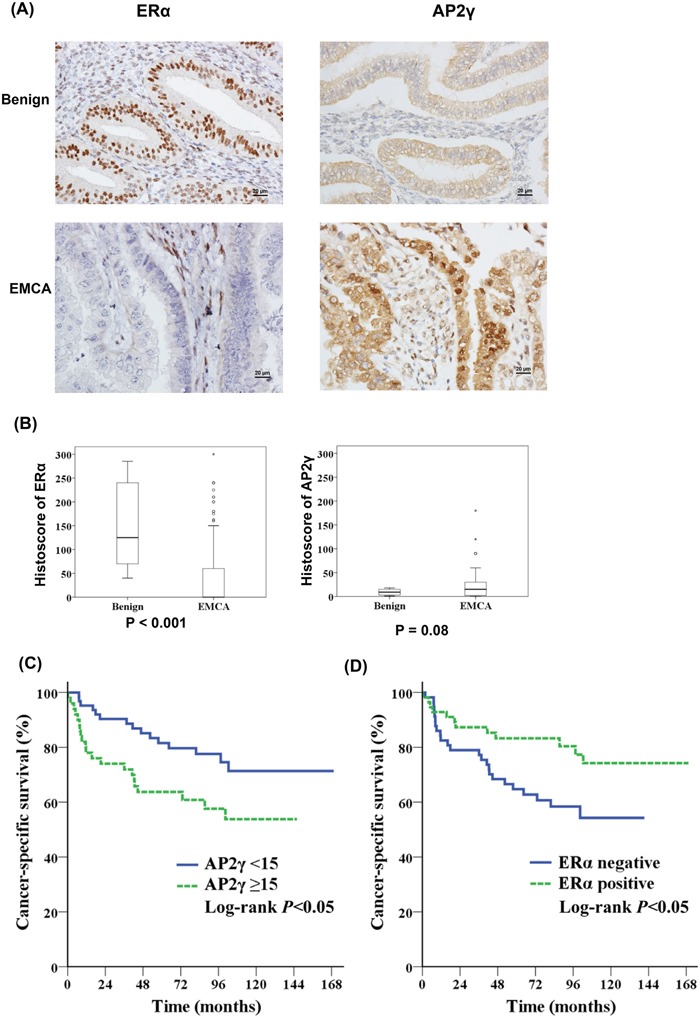
AP2γ expression levels are inversely associated with cancer-specific survival **A, B.** Protein levels of ERα (left panel) and AP2γ (right panel) in the endometrium were analyzed by immunohistochemistry. Normal endometrial tissues were obtained from patients who underwent hysterectomy for benign endometrial conditions (benign; n = 30) and endometrial cancer (n = 113). (A) Representative immunohistochemical staining of ERα (left panel) and AP2γ (right panel) in benign endometrial tissues (upper panel) and endometrial cancer (EMCA) (lower panel). (B) ERα (left panel) and AP2γ (right panel) staining was expressed with a histoscore (percentage of antibody-positive cells multiplied by their staining intensity). Kaplan-Meier curves indicated that **C.** patients with a high AP2γ histoscore (n = 50) had a lower cancer-specific survival than those with a low AP2γ histoscores (n = 63). **D.** ERα-negative (n = 57) patients had a lower cancer-specific survival than those who were ERα-positive (n = 56).

We calculated the histoscores for ERα, B23, and AP2γ in FFPE tissue samples from patients with benign endometrial conditions (n = 30) and endometrial cancer (n = 113). ERα histoscores were significantly higher in benign tissues than in endometrial cancer (P < 0.001), whereas an opposite pattern was observed for AP2γ histoscores (Figures 5A and 5B). Patients with endometrial cancer showing high AP2γ histoscores had worse outcomes than those with low AP2γ expression levels; opposite results were evident for ERα expression (Figures 5C and 5D). The B23 histoscore did not differ significantly between benign endometrial tissues and endometrial cancer specimens (data not shown).

## DISCUSSION

**Figure 6:**
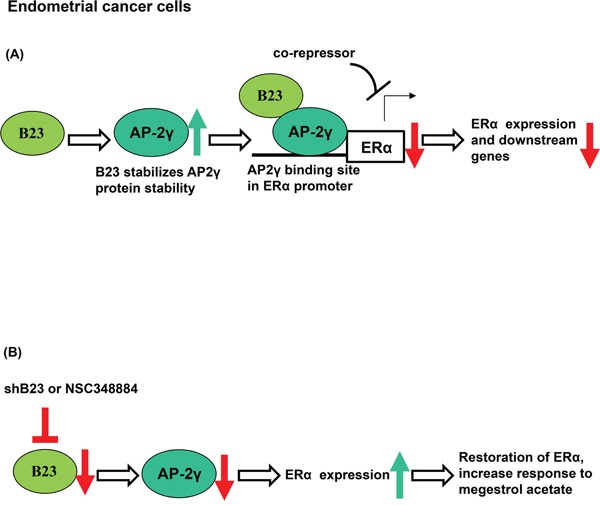
Schematic model of the mechanism by which B23 regulates ERα through AP2γ in endometrial cancer cells **A.** B23 promotes AP2γ protein stability and interacts with AP2γ as a co-repressor of the ERα promoter in endometrial cancer cells. ERα-activated genes were subsequently inhibited. **B.** Knockdown of B23 (either using shB23 or a B23 inhibitor) restores ERα expression, ultimately sensitizing hormone-refractory endometrial cancers to endocrine therapy.

Endometrial cancers expressing estrogen and progesterone receptors respond to hormonal therapy [38]. In contrast, a reduced expression of ERα has been associated with poor differentiation, advanced-stage tumors, disease recurrence, and adverse outcomes [39]. Here, we show for the first time that B23 suppression restores ERα expression in endometrial cancer. We also demonstrate that B23 forms a complex with AP-2γ and binds to the ERα promoter, ultimately acting as a co-repressor for ERα expression in endometrial cancer. Importantly, restoration of ERα expression through B23 inhibition sensitizes endometrial cancer to hormonal therapy (Figure 6).

In accordance with a previous study [40], continual passage in culture was associated with a loss of ERα expression in endometrial cancer cells. This phenomenon mimics the reduced ERα expression frequently observed in patients with advanced endometrial cancer, when unresponsiveness to hormonal therapy occurs commonly. Intratumor heterogeneity and subsequent clonal selection of receptor-negative cells may partly explain the loss of ERα expression during tumor recurrence [8]. Our data indicate that B23 or AP2γ suppression can restore ERα expression, suggesting their involvement in endometrial tumorigenesis.

In general, endometrioid adenocarcinomas are estrogen-dependent tumors [41]. A prolonged exposure to estrogens promotes the proliferation of human endometrial cancer cells through an increased B23 expression [27]. Our findings demonstrate that B23 can act as a transcriptional suppressor of ERα, ultimately inhibiting various ERα-targeted downstream genes (e.g., cathepsin D [42], EBAG9 [43], and TFF1/pS2 [44]). Another interesting observation is that patients with tumors expressing low AP2γ levels had better survival rates (Figure 5). AP2γ has been shown to play an essential role in hormone-related tumorigenesis in breast cancer [45, 46] and germ cell tumors [47, 48]. Interestingly, AP2γ and metastasis-associated protein 1 are involved in the epigenetic control of ERα transcription in breast malignancies [49] and portend a poor prognosis [50]. An increased expression of AP2γ has been also reported in advanced ovarian carcinomas [51].

The inhibitory effects of B23 and AP-2γ on ERα expression identified in endometrial cancer cells are similar to those observed in ERα-negative, MDA-MB-231, and Hs578T breast cancer cells (Supplementary Figure S2a). However, opposite results were obtained when ERα-positive MCF7 and T47D breast cancer cells were used (Supplementary Figure S2b). A forced expression of ERα has been shown to inhibit cell proliferation in ERα-negative MDA-MB-231 cells [52] as well as in a MCF7 ERα-negative subclone [53]. Furthermore, treatment with estradiol can reduce cell proliferation in ERα-negative but not in ERα-positive breast cancer cells expressing a recombinant ER [54]. The results of our study indicate that the role of B23 in the regulation of ERα expression differs significantly in endometrial and breast cancer cells, ultimately suggesting that AP-2γ function may be cell-specific and/or context-dependent (either acting as a transcriptional activator or repressor of ERα expression).

Both progesterone receptors and estrogen receptors have been actively investigated in relation to hormonal therapy response in patients with endometrial cancer. The results of the Gynecology Oncology Group Study (GOG) 81 trial have shown that the expression levels of progesterone receptors and estrogen receptors are highly correlated to each other in patients with advanced or recurrent endometrial cancer treated with progestins [55]. In addition, data from the parallel GOG 119 trial demonstrated that the estrogen receptor status is significantly related to clinical response and survival in patients who receive tamoxifen and medroxyprogesterone acetate [56]. Progestins and tamoxifen are currently utilized for hormonal therapy of patients with low-grade, early-stage endometrial cancer who are willing to preserve their fertility [57, 58]. Moreover, these drugs are used in combination with chemotherapy for patients with advanced or recurrent tumors expressing estrogen and/or progesterone receptors [59, 60]. It has been hypothesized that tamoxifen acts via estrogen receptors to increase progesterone receptors, ultimately sensitizing tumor cells to progestins [61, 62]. It is somehow intriguing that restoration of ERα without changing nuclear progesterone receptor levels renders cancer cells susceptible to megesterol acetate (Figure 4 and Supplementary Figure S6). These results further highlight the potential importance of recently reported membrane-bound progesterone receptors [63–65].

NSC348884 is a B23 inhibitor capable of disrupting the formation of B23 oligomers and suppressing their function in cancer cells [66]. Here, we show that inhibiting B23 with NSC348884 can restore ERα expression in human endometrial cancer cells as well asin a tumor xenograft model (Supplementary Figure S6). Taken together, our results suggest that restoration of ERα expression through the suppression of B23 or AP2γ can sensitize endometrial cancer cells to megestrol (Figures 4 and 6), potentially allowing patients with recurrent endometrial cancer to resume endocrine treatment.

### Conclusions

Our data indicate that B23 and AP2γ form a complex that acts as a transcriptional repressor and reduces ERα expression in endometrial cancer cells. Inhibition of B23 or AP2γ can restore ERα expression and may be useful for sensitizing hormone-refractory endometrial cancers to endocrine therapy. Further studies are warranted to shed more light on the therapeutic implications of our approach.

## MATERIALS AND METHODS

### Cultures

Ishikawa endometrial cancer cells were kindly provided by Dr. Nishida (National Hospital Organization, Kasumigaura Medical Center, Japan) [67]. The uterine serous carcinoma cell line ARK2 was obtained from Dr. Santin (Yale University, School of Medicine, New Haven, CT, USA) [68]. Ishikawa cells were grown in α-MEM medium containing 15% (v/v) fetal bovine serum (FBS). ARK2 cells were grown in RPMI-1640 medium containing 10% (v/v) FBS. RL95-2 endometrial cancer cells as well as Hs578T, MCF-7, and T47D breast cancer cells were cultured in Dulbecco's modified Eagle's medium supplemented with 10% FBS. MDA-MB-231 breast cancer cells were grown in L-15 medium supplemented with 10% FBS.

### Definition of cell passage

Ishikawa cells directly obtained from Dr. Nishida were considered as the first passage (n = 1) of the primary culture. Ishikawa cells were grown at 80% confluency with a subculturing ratio of 1:5 every 2−3 days. Low-passage cells were defined as those having less than 30 passages, whereas cells that had undergone more than 40 passages were considered as high-passage.

### Antibodies and reagents

Murine monoclonal antibodies raised against B23, Myc, and β-actin were purchased from Santa Cruz Biotechnology (Santa Cruz, CA, USA). Anti-ERα, anti-AP2γ, and anti-PR rabbit polyclonal antibodies were from Epitomics (Burlingame, CA, USA). Because AP2γ molecular size is similar to that of immunoglobulin heavy chains, a TrueBlot rabbit secondary antibody that recognizes only the native form of immunoglobulins (eBiosciences, San Diego, CA, USA) was used to avoid ambiguous signals in AP2γ immunoblotting experiments. All chemicals were purchased from Sigma (St. Louis, MO, USA) unless otherwise indicated.

### Estradiol treatment

Cells were cultured in steroid-depleted medium for 24 h in the absence of phenol red. The culture medium was then exposed to estradiol (E2) for 24 h. Negative controls were not exposed to E2.

### Western blot analysis

Western blot analysis was performed as previously described [27, 69]. In brief, cells were harvested and washed twice in phosphate-buffered saline (PBS), and then lysed in ice-cold RIPA lysis buffer [1% Triton X-100, 1% NP40, 0.1% SDS, 0.5% DOC, 20 mM Tris-HCl pH 7.4, 150 mM NaCl, and cocktail protease inhibitor (Sigma, St. Louis, MO, USA)] for 30 min. Lysates were boiled in 4× sample buffer dye (250 mM Tris-HCl, pH6.8, 8% SDS, 0.1% bromophenol blue, 40% glycerol, 400 mM β-mercaptoethanol) and subsequently subjected to 10% sodium dodecyl-sulfate polyacrylamide gel electrophoresis (SDS-PAGE). Proteins separated by SDS-PAGE were electrotransferred onto a Hybond-PVDF membrane (Amersham Pharmacia Biotech/GE Healthcare, Piscataway, NJ, USA). Blots were probed with appropriate primary and secondary antibodies. Bound antibodies were detected with an enhanced chemiluminescence system (ECL, Amersham Pharmacia Biotech/GE Healthcare).

### Quantitative real-time qPCR

Quantitative real-time qPCR (RT-qPCR) was performed with each triplicate RNA preparation. The housekeeping gene GAPDH was used for normalization. The primer sequences were as follows: ERα, 5′-GGAGGGCAGGGGTGAA-3′ (sense), 5′-GGCCAGGCTGTTCTTCTTAG-3′(antisense); B23, 5′- GGGGCTTTGAAATAACACCA-3′ (sense), 5′-GAAC CTTGCTACCACCTCCA-3′ (antisense); GAPDH, 5′-GGTATCGTGGAAGGACTCATGAC-3′ (sense), 5′-ATGCCAGTGAGCTTCCCGT-3′ (antisense); AP2γ, 5′- ACTGTCCCGACCTGAATGCT-3′ (sense), 5′-CGA TTTGGCTCTTCTGAGAACA-3′(antisense); cathepsin D, 5′-GTACATGATCCCCTGTGAGAAGGT-3′ (sense), 5′-GGGACAGCTTGTAGCCTTTGC-3′ (antisense); EBAG9, 5′-GATGCACCCACCAGTGTAAAGA-3′ (sense), 5′-AGTCAGGTTCCAGTTGTTCCAAAG (antisense); TFF1/pS2, 5′-ACATGGAAGGATTTGC TGATA -3′ (sense), 5′-TTCCGGCCATCTCTCACTAT -3′ (antisense). The amplification conditions were as follows: initial denaturation at 95°C for 10 min, followed by 45 cycles of 95°C for 15 sec and 60°C for 1 min. All reactions were performed with an ABI PRISM 7900 HT instrument (Applied Biosystems, Foster City, CA, USA). A mean cycle of threshold (Ct) value for each duplicate measurement was calculated.

### Promoter deletion construct and site-directed mutagenesis

We then analyzed the transcriptional properties of the ERα promoter reporter plasmid. To this aim, we obtained a PCR fragment using a pair of primers that covered the sequence between positions −1994 and +210 of the promoter. The primer sequences used for the ERα promoter fragment were as follows: 5′- CTCGCACATGCGAGCACATT-3′ (sense), 5′-GCTCGTTCCCTTGGATCTGA-3′ (antisense). The promoter fragment was digested and inserted in both sense and antisense orientations into the *Kpn*I and *Xho*I restriction sites of the pGL3 Basic Vector (Promega, Madison, WI, USA). The deletion constructs were cloned using the *Kpn*I and *Xho*I sites to generate the region between positions −384 and +210 of the promoter. Mutations were introduced into a putative AP2γ recognition site in the ERα promoter region using site-directed mutagenesis using a two-step PCR. The forward primer for mutant AP2γ was 5′-CCTTCTGCAATGCAAGG-3′.

### Reporter gene assay

Cell extracts for the ERα promoter reporter assay were obtained with 1× Reporter lysis buffer (Promega). The reporter/luciferase activity of ERα was measured with a luciferase reporter assay (Promega) after normalization with the corresponding β-galactosidase activity.

### Immunoprecipitation

Cells were harvested and washed twice in ice-cold PBS. Cell pellets were then resuspended in ice-cold WCE lysis buffer (20 mM HEPES, 10% glycerol, 0.5% Triton X-100, 0.2 M sodium chloride, 1 mM EDTA, 1 mM EGTA, and protease inhibitor cocktail) for 30 min and centrifuged at 12000 rpm at 4°C for 30 min. Equal amounts of protein from each cell extract were incubated with the antibodies of interest (2 μg) at 4°C for 2 h. Immune complexes were captured by incubation with protein G-sepharose (30 μL; Upstate Biotechnology, Lake Placid, NY, USA) for 2 h at 4°C under rotation. The protein G-antigen-antibody complexes were washed four times with the WCE lysis buffer, and boiled in 4× sample buffer dye (250 mM Tris-HCl, pH 6.8, 8% SDS, 0.1% bromophenol blue, 40% glycerol, 400 mM β-mercaptoethanol) before PAGE and Western blot analysis.

### Chromatin immunoprecipitation

The protocol for chromatin immunoprecipitation (ChIP) has been previously described [35, 70]. Crosslinked chromatin was pre-cleared and incubated with 2 μg antibody (B23 or AP2γ) and rotated at 4°C overnight. A mock immunoprecipitation with IgG was used as a negative control. After extensive washing, the immune complexes were decrosslinked and treated with proteinase K. Bound DNA in B23 or AP2γ ChIP was extracted, purified, and subjected to PCR analysis using the following primers targeting the promoter sequence of ERα gene: 5′-GCCTCTAACCTCGGGCTGTGCTCTT-3′ (sense) and 5′-GCTCGTTCCCTTGGATCTGA-3′ (antisense). Bound DNA for anti- B23 or anti-AP2γ ChIP experiments was subjected to real-time qPCR analysis.

### Generation of short hairpin RNA

With the goal of establishing a plasmid-based dsRNAi system for targeting endogenous B23 and AP2γ, annealed oligonucleotides corresponding to a partial sequence were designed and ligated to the expression vector pSuper.neo+GFP (OligoEngine, Seattle, WA, USA). The cDNA sequence of the targeted mRNA region for the B23 and Luc genes were as follows: B23: 5′-TGATGAAAATGAGCACCAGTT-3′; AP2γ: 5′-AUCAGCUGGACUCUGGUC UCC AGGG-3′; Luc: GTAGGAGTAGTGAAAGGCC-3′.

### Cell transfection and generation of stable clones

We established a plasmid-based dsRNAi system targeting endogenous B23 and AP2γ by incubating Lipofectamine 2000 reagent (Invitrogen/Life Technologies, Carlsbad, CA, USA) with Opti-MEM medium (Invitrogen/Life Technologies) for 5 min at room temperature. Plasmids were added to Lipofectamine 2000 mixture and incubated at room temperature for 30 min to promote the proper formation of transfection complexes. Transfection mixtures were then added to cells in Opti-MEM medium. After 6 h of incubation in a CO_2_ incubator at 37°C, the DNA-containing medium was replaced by fresh medium containing 10% serum. We established stable clones by culturing transfected cells in culture media with G418 (0.8 mg/mL) at 48 h after transfection. After exposure to G418 for 3 weeks, individual clones were selected for culture and subsequently assayed for B23 expression. Transfected cells were maintained in culture medium supplemented with G418 (0.2 mg/mL). Stable clones were established by culturing transfected cells in culture media containing G418 (0.8 mg/mL) at 48 h after transfection. After selection with G418 for 3 weeks, individual clones were cultured and subsequently assayed for B23 levels and AP2γ expression. All transfections were maintained in culture medium supplemented with G418 (0.2 mg/mL).

### Viability assays

Approximately 5000 cells were seeded onto the wells of a 96-well culture plate for 24 h. The original medium was replaced by a steroid-depleted medium lacking phenol red (Invitrogen/Life Technologies) with either megestrol acetate or vehicle for 72 h. We then established a colorimetric MTT assay. To this aim, MTT (5 mg/mL, 25 μL) was added into the wells containing treated cells. After 4 h, the supernatant was discarded and DMSO (100 μL) was added to each well. The optical density of the mixture was measured at 570 nm with an ELISA microplate reader (PerkinElmer VICTOR 2; GMI, Ramsey, MN, USA).

### Tissue specimens

Normal endometrial tissues were obtained from patients who underwent hysterectomy for benign gynecological diseases (n = 30). Malignant tissue samples were collected from patients undergoing surgery for endometrial cancer (n = 113). Clinical data were retrieved from the databank of the Division of Gynecologic Oncology, Chang Gung Memorial Hospital, Taiwan. The study protocol was approved by the Institutional Review Board of the Chang Gung Memorial Hospital, Taiwan (IRB#101-4162B, IRB#102-3837C).

### Immunohistochemistry

The immunohistochemistry (IHC) protocol has been previously described in detail [27, 69]. Formalin-fixed, paraffin-embedded tissue slices (4-μm thick) were deparaffinized in xylene and rehydrated through a series of graded ethanol baths. Sections were then stained with a mouse anti-human B23 monoclonal antibody (Santa Cruz Biotechnology), a rabbit anti-human AP2γ polyclonal antibody, or a rabbit anti-human ERα polyclonal antibody (Santa Cruz Biotechnology) using an automated IHC stainer with the Ventana Basic DAB Detection kit (Tucson, AZ, USA). Counterstaining was performed with hematoxylin. A global immunohistochemical score (histoscore) was calculated as the percentage of positive cells multiplied by the staining intensity (0 = negative, 1 = weak, 2 = moderate, 3 = strong). The histoscore ranged from 0 to 300 (100% multiplied by 3) [27]. Part of the IHC data for both benign and malignant tissues was retrieved from our previously published study [27].

### Animals and treatment

Six-week-old female BALC/c nude mice were obtained from the National Laboratory Animal Center, Taiwan. All animal procedures were approved by the Animal Care Committee of the Institutional Review Board, Chang Gung Memorial Hospital (2012050903). For the purpose of NSC348884 treatment, ARK2 cells were harvested, washed, and resuspended in Hanks' balanced salt solution (HBSS) at a concentration of 10^7^ cells/mL. Tumors were established by subcutaneous inoculation of a cell suspension (100 μL) into the lateral hind leg of nude mice (aged 6−8 weeks). After 20 days, NSC348884 (0.4 mg/100 μL) was weekly administered to tumor-bearing mice and control animals. For the purpose of megestrol acetate treatment, Ishikawa cells and shLuc, shB23, and shAP2γ stable lines were harvested, washed, and resuspended in HBSS at a concentration of 10^7^ cells/mL. Tumors were established by subcutaneous inoculation of a cell suspension (100 μL) into the lateral hind leg of nude mice (aged 6−8 weeks). After 83 days, megestrol acetate (10 mg/kg) was administered (5 days per week) to tumor-bearing mice and control animals. During the treatment course, tumor growth was monitored on a weekly basis. Tumor volumes (cm^3^) in tumor-bearing mice were determined with an *in vivo* assay for tumor mass. Upon completion of the experiments, tumors were excised and fixed in formaldehyde. Tissue samples were stained with hematoxylin and eosin. Tumor tissue extracts for Western blot were also obtained.

### Statistical analysis

Continuous variables were analyzed with the Mann-Whitney *U* test. All calculations were performed with the SPSS 17.0 statistical package (SPSS Inc., Chicago, IL, USA). Two-tailed *P* values <0.05 were considered statistically significant.

## SUPPLEMENTARY FIGURES



## References

[1] Jemal A, Bray F, Center MM, Ferlay J, Ward E, Forman D (2011). Global cancer statistics. CA Cancer J Clin.

[2] (2012). Cancer Registry Annual Report 2010.

[3] Creasman WT, Soper JT, McCarty KS, McCarty KS, Hinshaw W Sr, Clarke-Pearson DL (1985). Influence of cytoplasmic steroid receptor content on prognosis of early stage endometrial carcinoma. Am J Obstet Gynecol.

[4] Creasman WT, Miller DS, Di Saia PJ, Creasman WT, Mannel RS, McMeekin DS, Mutch DG (2012). Adenocarcinoma of the uterine corpus. Clinical Gynecologic Oncology.

[5] Sutton GP, Geisler HE, Stehman FB, Young PC, Kimes TM, Ehrlich CE (1989). Features associated with survival and disease-free survival in early endometrial cancer. Am J Obstet Gynecol.

[6] Ehrlich CE, Young PC, Stehman FB, Sutton GP, Alford WM (1988). Steroid receptors and clinical outcome in patients with adenocarcinoma of the endometrium. Am J Obstet Gynecol.

[7] Kauppila A (1989). Oestrogen and progestin receptors as prognostic indicators in endometrial cancer A review of the literature. Acta Oncol.

[8] Kuukasjarvi T, Kononen J, Helin H, Holli K, Isola J (1996). Loss of estrogen receptor in recurrent breast cancer is associated with poor response to endocrine therapy. J Clin Oncol.

[9] Spataro V, Goldhirsch A (1997). Breast cancer: relevance of estrogen receptor determination at relapse. J Clin Oncol.

[10] Hua S, Kittler R, White KP (2009). Genomic antagonism between retinoic acid and estrogen signaling in breast cancer. Cell.

[11] Ross-Innes CS, Stark R, Holmes KA, Schmidt D, Spyrou C, Russell R, Massie CE, Vowler SL, Eldridge M, Carroll JS (2010). Cooperative interaction between retinoic acid receptor-alpha and estrogen receptor in breast cancer. Genes Dev.

[12] Hurtado A, Holmes KA, Geistlinger TR, Hutcheson IR, Nicholson RI, Brown M, Jiang J, Howat WJ, Ali S, Carroll JS (2008). Regulation of ERBB2 by oestrogen receptor-PAX2 determines response to tamoxifen. Nature.

[13] Eeckhoute J, Keeton EK, Lupien M, Krum SA, Carroll JS, Brown M (2007). Positive cross-regulatory loop ties GATA-3 to estrogen receptor alpha expression in breast cancer. Cancer Res.

[14] Holmes KA, Song JS, Liu XS, Brown M, Carroll JS (2008). Nkx3-1 and LEF-1 function as transcriptional inhibitors of estrogen receptor activity. Cancer Res.

[15] Caizzi L, Ferrero G, Cutrupi S, Cordero F, Ballare C, Miano V, Reineri S, Ricci L, Friard O, Testori A, Cora D, Caselle M (2014). Genome-wide activity of unliganded estrogen receptor-alpha in breast cancer cells. Proc Natl Acad Sci U S A.

[16] Tan SK, Lin ZH, Chang CW, Varang V, Chng KR, Pan YF, Yong EL, Sung WK, Cheung E (2011). AP-2gamma regulates oestrogen receptor-mediated long-range chromatin interaction and gene transcription. EMBO J.

[17] Eckert D, Buhl S, Weber S, Jager R, Schorle H (2005). The AP-2 family of transcription factors. Genome Biol.

[18] Pellikainen JM, Kosma VM (2007). Activator protein-2 in carcinogenesis with a special reference to breast cancer—a mini review. Int J Cancer.

[19] McPherson LA, Baichwal VR, Weigel RJ (1997). Identification of ERF-1 as a member of the AP2 transcription factor family. Proc Natl Acad Sci U S A.

[20] Bosher JM, Williams T, Hurst HC (1995). The developmentally regulated transcription factor AP-2 is involved in c-erbB-2 overexpression in human mammary carcinoma. Proc Natl Acad Sci U S A.

[21] Williams CM, Scibetta AG, Friedrich JK, Canosa M, Berlato C, Moss CH, Hurst HC (2009). AP-2gamma promotes proliferation in breast tumour cells by direct repression of the CDKN1A gene. EMBO J.

[22] Ning C, Xie B, Zhang L, Li C, Shan W, Yang B, Luo X, Gu C, He Q, Jin H, Chen X, Zhang Z (2016). Infiltrating Macrophages Induce ERalpha Expression through an IL17A-mediated Epigenetic Mechanism to Sensitize Endometrial Cancer Cells to Estrogen. Cancer Res.

[23] Yang S, Xiao X, Jia Y, Liu X, Zhang Y, Wang X, Winters CJ, Devor EJ, Meng X, Thiel KW, Leslie KK (2014). Epigenetic modification restores functional PR expression in endometrial cancer cells. Curr Pharm Des.

[24] Yang X, Phillips DL, Ferguson AT, Nelson WG, Herman JG, Davidson NE (2001). Synergistic activation of functional estrogen receptor (ER)-alpha by DNA methyltransferase and histone deacetylase inhibition in human ER-alpha-negative breast cancer cells. Cancer Res.

[25] Li Y, Meeran SM, Patel SN, Chen H, Hardy TM, Tollefsbol TO (2013). Epigenetic reactivation of estrogen receptor-alpha (ERalpha) by genistein enhances hormonal therapy sensitivity in ERalpha-negative breast cancer. Mol Cancer.

[26] Okuwaki M (2008). The structure and functions of NPM1/Nucleophsmin/B23 a multifunctional nucleolar acidic protein. J Biochem.

[27] Chao A, Lin CY, Tsai CL, Hsueh S, Lin YY, Lin CT, Chou HH, Wang TH, Lai CH, Wang HS (2013). Estrogen stimulates the proliferation of human endometrial cancer cells by stabilizing nucleophosmin/B23 (NPM/B23). J Mol Med (Berl).

[28] Shields LB, Gercel-Taylor C, Yashar CM, Wan TC, Katsanis WA, Spinnato JA, Taylor DD (1997). Induction of immune responses to ovarian tumor antigens by multiparity. J Soc Gynecol Investig.

[29] Pianta A, Puppin C, Franzoni A, Fabbro D, Di Loreto C, Bulotta S, Deganuto M, Paron I, Tell G, Puxeddu E, Filetti S, Russo D (2010). Nucleophosmin is overexpressed in thyroid tumors. Biochem Biophys Res Commun.

[30] Tanaka M, Sasaki H, Kino I, Sugimura T, Terada M (1992). Genes preferentially expressed in embryo stomach are predominantly expressed in gastric cancer. Cancer Res.

[31] Nozawa Y, Van Belzen N, Van der Made AC, Dinjens WN, Bosman FT (1996). Expression of nucleophosmin/B23 in normal and neoplastic colorectal mucosa. J Pathol.

[32] Subong EN, Shue MJ, Epstein JI, Briggman JV, Chan PK, Partin AW (1999). Monoclonal antibody to prostate cancer nuclear matrix protein (PRO:4-216) recognizes nucleophosmin/B23. Prostate.

[33] Tsui KH, Cheng AJ, Chang P, Pan TL, Yung BY (2004). Association of nucleophosmin/B23 mRNA expression with clinical outcome in patients with bladder carcinoma. Urology.

[34] Chen CL, Tsui KH, Lin CY, Chang PL, Tang P, Yung B (2007). Can probability of genetic mutation be an indicator of clinical relevance?. Genomics.

[35] Lin CY, Liang YC, Yung BY (2006). Nucleophosmin/B23 regulates transcriptional activation of E2F1 via modulating the promoter binding of NF-kappaB E2F1 and pRB. Cell Signal.

[36] Schuur ER, McPherson LA, Yang GP, Weigel RJ (2001). Genomic structure of the promoters of the human estrogen receptor-alpha gene demonstrate changes in chromatin structure induced by AP2gamma. J Biol Chem.

[37] Liu H, Tan BC, Tseng KH, Chuang CP, Yeh CW, Chen KD, Lee SC, Yung BY (2007). Nucleophosmin acts as a novel AP2alpha-binding transcriptional corepressor during cell differentiation. EMBO Rep.

[38] Hacker NF, Friedlander M, Berek JS, Hacker NF (2009). Uterine cancer. Berek and Hacker's Gynecologic Oncology.

[39] Jongen V, Briet J, de Jong R, ten Hoor K, Boezen M, van der Zee A, Nijman H, Hollema H (2009). Expression of estrogen receptor-alpha and -beta and progesterone receptor-A and -B in a large cohort of patients with endometrioid endometrial cancer. Gynecol Oncol.

[40] Lesmeister MJ, Jorgenson RL, Young SL, Misfeldt ML (2005). 17Beta-estradiol suppresses TLR3-induced cytokine and chemokine production in endometrial epithelial cells. Reprod Biol Endocrinol.

[41] Hecht JL, Mutter GL (2006). Molecular and pathologic aspects of endometrial carcinogenesis. J Clin Oncol.

[42] Augereau P, Miralles F, Cavailles V, Gaudelet C, Parker M, Rochefort H (1994). Characterization of the proximal estrogen-responsive element of human cathepsin D gene. Mol Endocrinol.

[43] Suzuki T, Inoue S, Kawabata W, Akahira J, Moriya T, Tsuchiya F, Ogawa S, Muramatsu M, Sasano H (2001). EBAG9/RCAS1 in human breast carcinoma: a possible factor in endocrine-immune interactions. Br J Cancer.

[44] Koshiyama M, Yoshida M, Konishi M, Takemura M, Yura Y, Matsushita K, Hayashi M, Tauchi K (1997). Expression of pS2 protein in endometrial carcinomas: correlation with clinicopathologic features and sex steroid receptor status. Int J Cancer.

[45] Jager R, Werling U, Rimpf S, Jacob A, Schorle H (2003). Transcription factor AP-2gamma stimulates proliferation and apoptosis and impairs differentiation in a transgenic model. Mol Cancer Res.

[46] Orso F, Cottone E, Hasleton MD, Ibbitt JC, Sismondi P, Hurst HC, De Bortoli M (2004). Activator protein-2gamma (AP-2gamma) expression is specifically induced by oestrogens through binding of the oestrogen receptor to a canonical element within the 5′-untranslated region. Biochem J.

[47] Hoei-Hansen CE, Nielsen JE, Almstrup K, Sonne SB, Graem N, Skakkebaek NE, Leffers H, Rajpert-De Meyts E (2004). Transcription factor AP-2gamma is a developmentally regulated marker of testicular carcinoma in situ and germ cell tumors. Clin Cancer Res.

[48] Pauls K, Jager R, Weber S, Wardelmann E, Koch A, Buttner R, Schorle H (2005). Transcription factor AP-2gamma a novel marker of gonocytes and seminomatous germ cell tumors. Int J Cancer.

[49] Kang HJ, Lee MH, Kang HL, Kim SH, Ahn JR, Na H, Na TY, Kim YN, Seong JK, Lee MO (2014). Differential regulation of estrogen receptor alpha expression in breast cancer cells by metastasis-associated protein 1. Cancer Res.

[50] Zhao C, Yasui K, Lee CJ, Kurioka H, Hosokawa Y, Oka T, Inazawa J (2003). Elevated expression levels of NCOA3 TOP1 and TFAP2C in breast tumors as predictors of poor prognosis. Cancer.

[51] Odegaard E, Staff AC, Kaern J, Florenes VA, Kopolovic J, Trope CG, Abeler VM, Reich R, Davidson B (2006). The AP-2gamma transcription factor is upregulated in advanced-stage ovarian carcinoma. Gynecol Oncol.

[52] Garcia M, Derocq D, Freiss G, Rochefort H (1992). Activation of estrogen receptor transfected into a receptor-negative breast cancer cell line decreases the metastatic and invasive potential of the cells. Proc Natl Acad Sci U S A.

[53] Oesterreich S, Zhang P, Guler RL, Sun X, Curran EM, Welshons WV, Osborne CK, Lee AV (2001). Re-expression of estrogen receptor alpha in estrogen receptor alpha-negative MCF-7 cells restores both estrogen and insulin-like growth factor-mediated signaling and growth. Cancer Res.

[54] Zajchowski DA, Sager R, Webster L (1993). Estrogen inhibits the growth of estrogen receptor-negative but not estrogen receptor-positive human mammary epithelial cells expressing a recombinant estrogen receptor. Cancer Res.

[55] Thigpen JT, Brady MF, Alvarez RD, Adelson MD, Homesley HD, Manetta A, Soper JT, Given FT (1999). Oral medroxyprogesterone acetate in the treatment of advanced or recurrent endometrial carcinoma: a dose-response study by the Gynecologic Oncology Group. J Clin Oncol.

[56] Singh M, Zaino RJ, Filiaci VJ, Leslie KK (2007). Relationship of estrogen and progesterone receptors to clinical outcome in metastatic endometrial carcinoma: a Gynecologic Oncology Group Study. Gynecol Oncol.

[57] Lai CH, Hsueh S, Chao AS, Soong YK (1994). Successful pregnancy after tamoxifen and megestrol acetate therapy for endometrial carcinoma. Br J Obstet Gynaecol.

[58] Wang CJ, Chao A, Yang LY, Hsueh S, Huang YT, Chou HH, Chang TC, Lai CH (2014). Fertility-preserving treatment in young women with endometrial adenocarcinoma: a long-term cohort study. Int J Gynecol Cancer.

[59] Bevis KS, Kilgore LC, Alvarez RD, Straughn JM, Leath CA (2014). Combination therapy with paclitaxel, carboplatin and megestrol acetate for the management of advanced stage or recurrent carcinoma of the endometrium: a phase II study. J Reprod Med.

[60] Kokka F, Brockbank E, Oram D, Gallagher C, Bryant A (2010). Hormonal therapy in advanced or recurrent endometrial cancer. Cochrane Database Syst Rev.

[61] Whitney CW, Brunetto VL, Zaino RJ, Lentz SS, Sorosky J, Armstrong DK, Lee RB (2004). Phase II study of medroxyprogesterone acetate plus tamoxifen in advanced endometrial carcinoma: a Gynecologic Oncology Group study. Gynecol Oncol.

[62] Fiorica JV, Brunetto VL, Hanjani P, Lentz SS, Mannel R, Andersen W, Gynecologic Oncology Group s (2004). Phase II trial of alternating courses of megestrol acetate and tamoxifen in advanced endometrial carcinoma: a Gynecologic Oncology Group study. Gynecol Oncol.

[63] Salazar M, Lerma-Ortiz A, Hooks GM, Ashley AK, Ashley RL (2016). Progestin-mediated activation of MAPK and AKT in nuclear progesterone receptor negative breast epithelial cells: The role of membrane progesterone receptors. Gene.

[64] Xie M, Zhu X, Liu Z, Shrubsole M, Varma V, Mayer IA, Dai Q, Chen Q, You S (2012). Membrane progesterone receptor alpha as a potential prognostic biomarker for breast cancer survival: a retrospective study. PLoS One.

[65] Neubauer H, Yang Y, Seeger H, Fehm T, Cahill MA, Tong X, Ruan X, Mueck AO (2011). The presence of a membrane-bound progesterone receptor sensitizes the estradiol-induced effect on the proliferation of human breast cancer cells. Menopause.

[66] Qi W, Shakalya K, Stejskal A, Goldman A, Beeck S, Cooke L, Mahadevan D (2008). NSC348884 a nucleophosmin inhibitor disrupts oligomer formation and induces apoptosis in human cancer cells. Oncogene.

[67] Nishida M (2002). The Ishikawa cells from birth to the present. Hum Cell.

[68] Zhao S, Choi M, Overton JD, Bellone S, Roque DM, Cocco E, Guzzo F, English DP, Varughese J, Gasparrini S, Bortolomai I, Buza N (2013). Landscape of somatic single-nucleotide and copy-number mutations in uterine serous carcinoma. Proc Natl Acad Sci U S A.

[69] Wang TH, Chao A, Tsai CL, Chang CL, Chen SH, Lee YS, Chen JK, Lin YJ, Chang PY, Wang CJ, Chao AS, Chang SD (2010). Stress-induced phosphoprotein 1 as a secreted biomarker for human ovarian cancer promotes cancer cell proliferation. Mol Cell Proteomics.

[70] Lin CY, Tan BC, Liu H, Shih CJ, Chien KY, Lin CL, Yung BY (2010). Dephosphorylation of nucleophosmin by PP1beta facilitates pRB binding and consequent E2F1-dependent DNA repair. Mol Biol Cell.

