# Supplementary material for: Nucleophosmin/B23 is a negative regulator of estrogen receptor α expression via AP2γ in endometrial cancer cells
Source: Oncotarget. 2016 Aug 4;7(37):60038–52. doi: 10.18632/oncotarget.11048 (PMC5312367; doi:10.18632/oncotarget.11048)
Supplement: Supplementary file 1 [file oncotarget-07-60038-s001.pdf]

Nucleophosmin/B23 is a negative regulator of estrogen receptor α expression via AP2γ in endometrial cancer cells

SUPPLEMENTARY FIGURES

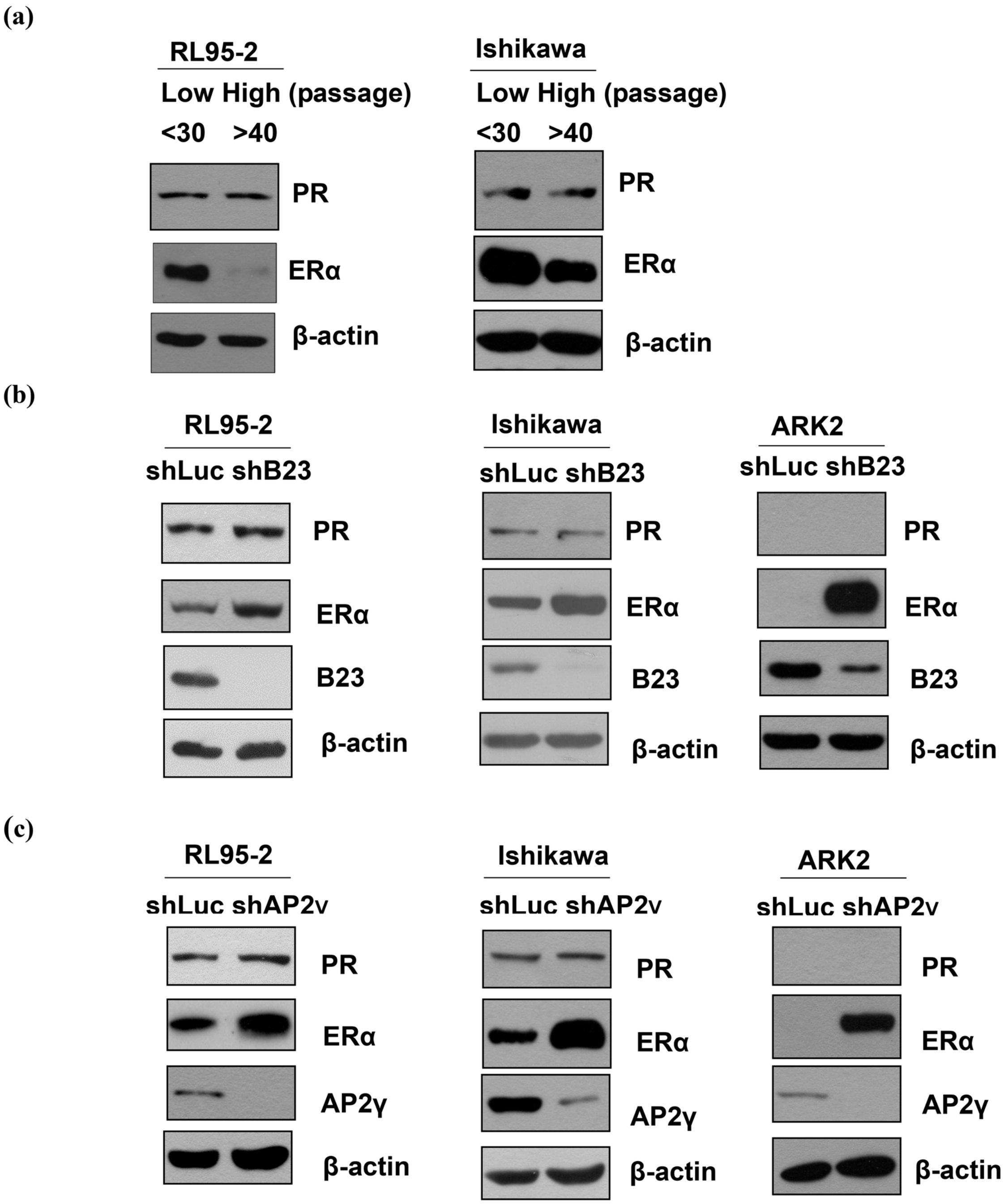

Supplementary Figure S1: PR expression did not affect by different passage or shRNA for B23 and AP2γ. a. RL95-2 and Ishikawa endometrial cancer cells with early passage (n <30) and late passage (n > 40) were subcultured in medium containing phenol-red every 2-3 days. Equal amounts of protein lysates were separated by SDS-PAGE and subjected to immunoblotting with antibodies for PR, ERα, AP2γ, B23 and β-actin. **b, c.** RL95-2, Ishikawa cells or ARK2 with shLuc, shB23 and shAP2γ stable lines were harvested, and equal amounts of protein lysates were separated by SDS-PAGE and subjected to immunoblotting with antibodies for PR, ERα, AP2γ, B23 and β-actin. β-actin was used to confirm equal protein inputs in all lanes.

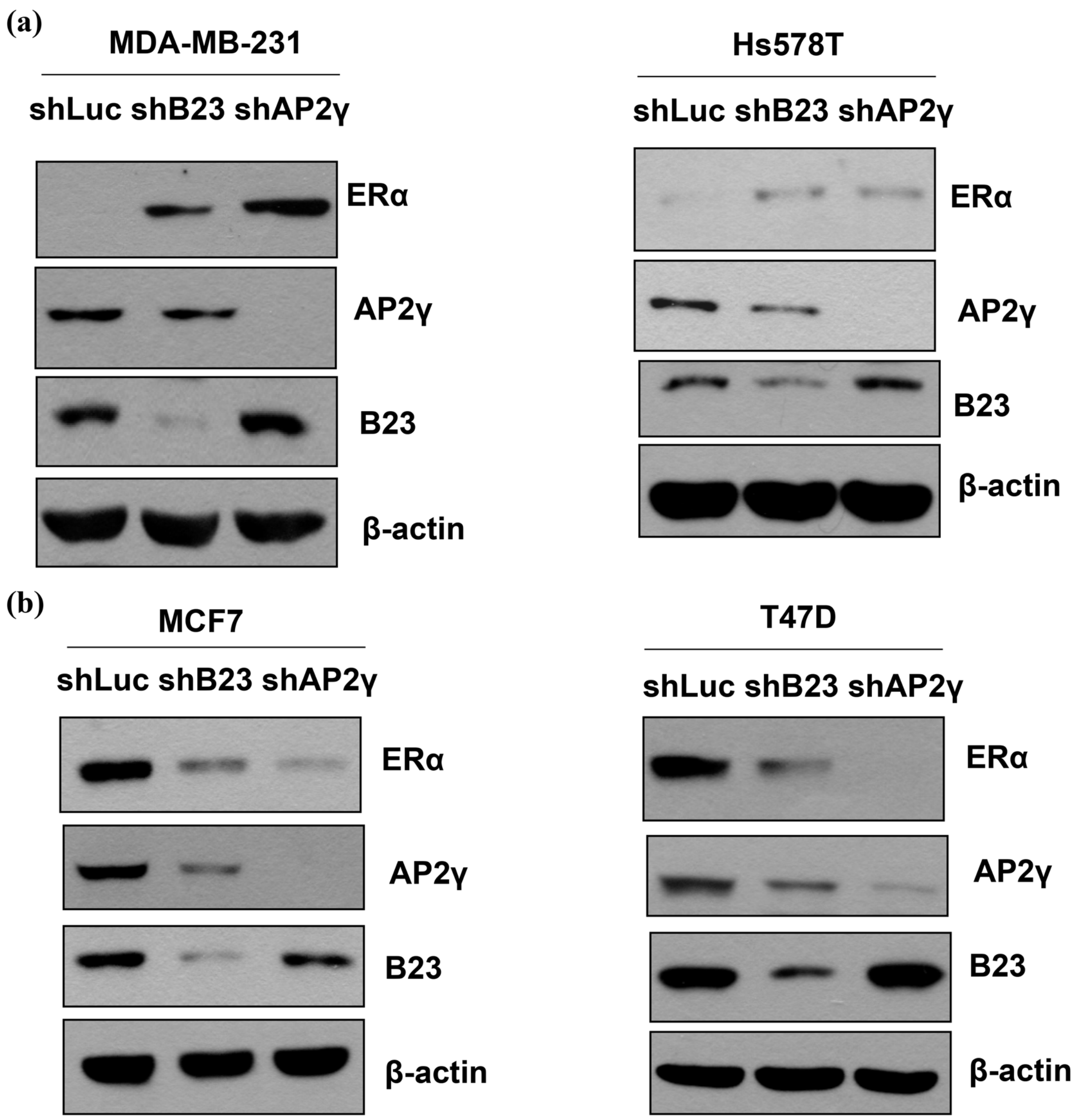

Supplementary Figure S2: B23 and AP2γ play different roles in ERα expression in various breast cancer cells. a. ERα-negative MDA-MB-231 and Hs578T breast cancer cells were transiently transfected with shLuc, shB23 or shAP2γ for 72 h and were immunoblotted with the indicated antibodies. **b.** ERα-positive MCF7 and T47D breast cancer cells were transiently transfected with shLuc, shB23 or shAP2γ for 72 h and were immunoblotted with the indicated antibodies. β-actin was used to confirm equal protein inputs in all lanes.

(a)

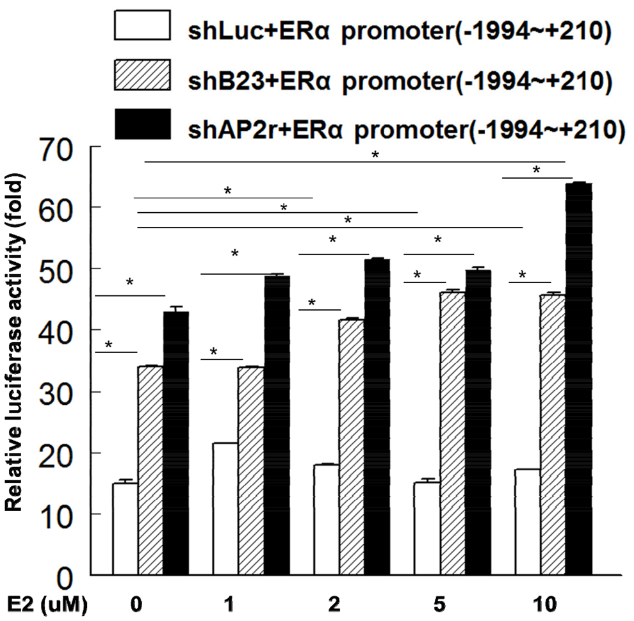

(b)

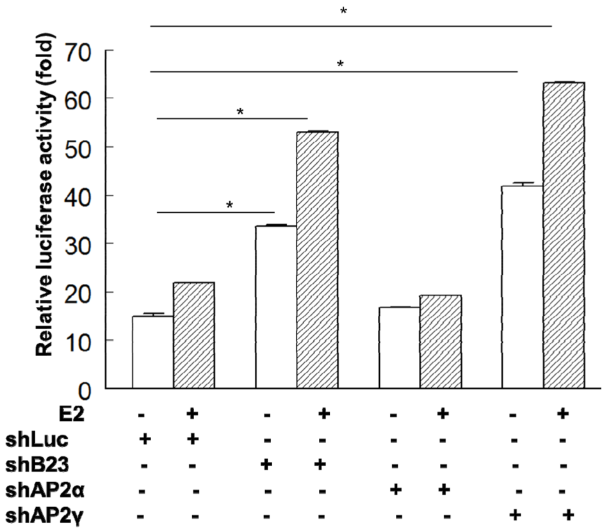

Supplementary Figure S3: Knockdown B23 or AP2γ enhances sensitivity of the ERα promoter activity to estradiol. a. ARK2 cells were transiently co-transfected with the ERα promoter and shLuc, shB23, shAP2γ for 24h and treated with/without different dose of E2 for additional 24h. Herein, shAP2α was used as a contrast to demonstrate the effect of knocking down AP2γ. **b.** ARK2 cells were transiently co-transfected with the ERα promoter and shLuc, shB23, shAP2α, shAP2γ for 24h and treated with/without E2 (1uM) for additional 24h Protein lysates were then assayed for luciferase and β-galactosidase activities. An approximate 15-fold increase in the ERα-promoter (-1994~+210) reporter activity was observed when compared with the empty pGL3-promoter vector, where the activity was set as 1. The relative promoter activity was normalized to that of β-galactosidase. * $P < 0.05$ compared with controls.

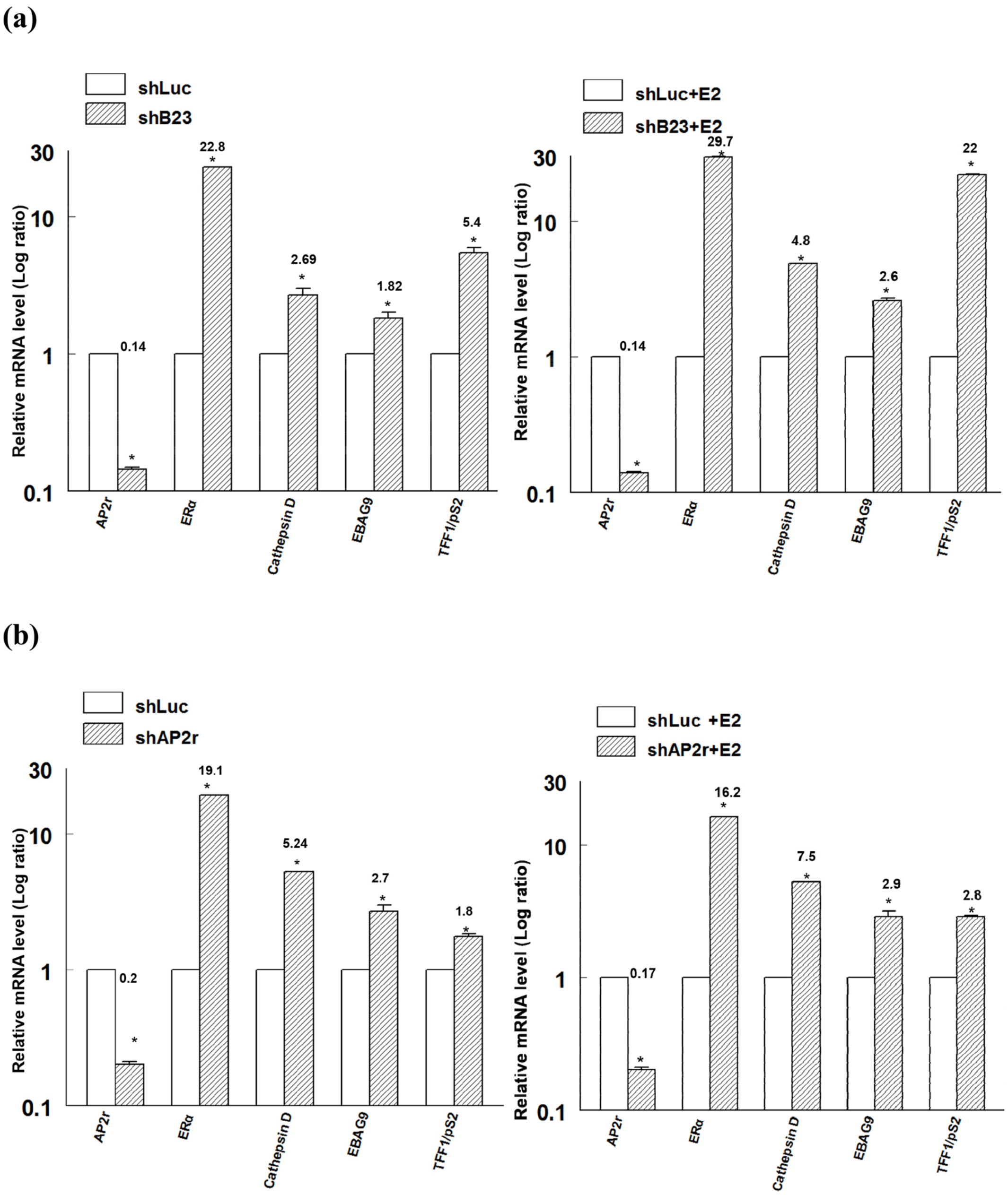

Supplementary Figure S4: Knockdown B23 or AP2γ enhances sensitivity of the ERα downstream genes to estradiol treatment. a, b. ARK2 cells were transiently transfected with shLuc or shB23 or shAP2γ for 48h and treated with/without 1uM E2 for additional 24h. The resulting RNAs of B23, AP2γ, ERα, cathepsin D, EBAG9, and TFF1/pS2 were analyzed with real-time qPCR using the designated primers. *$P < 0.05$ compared with controls.

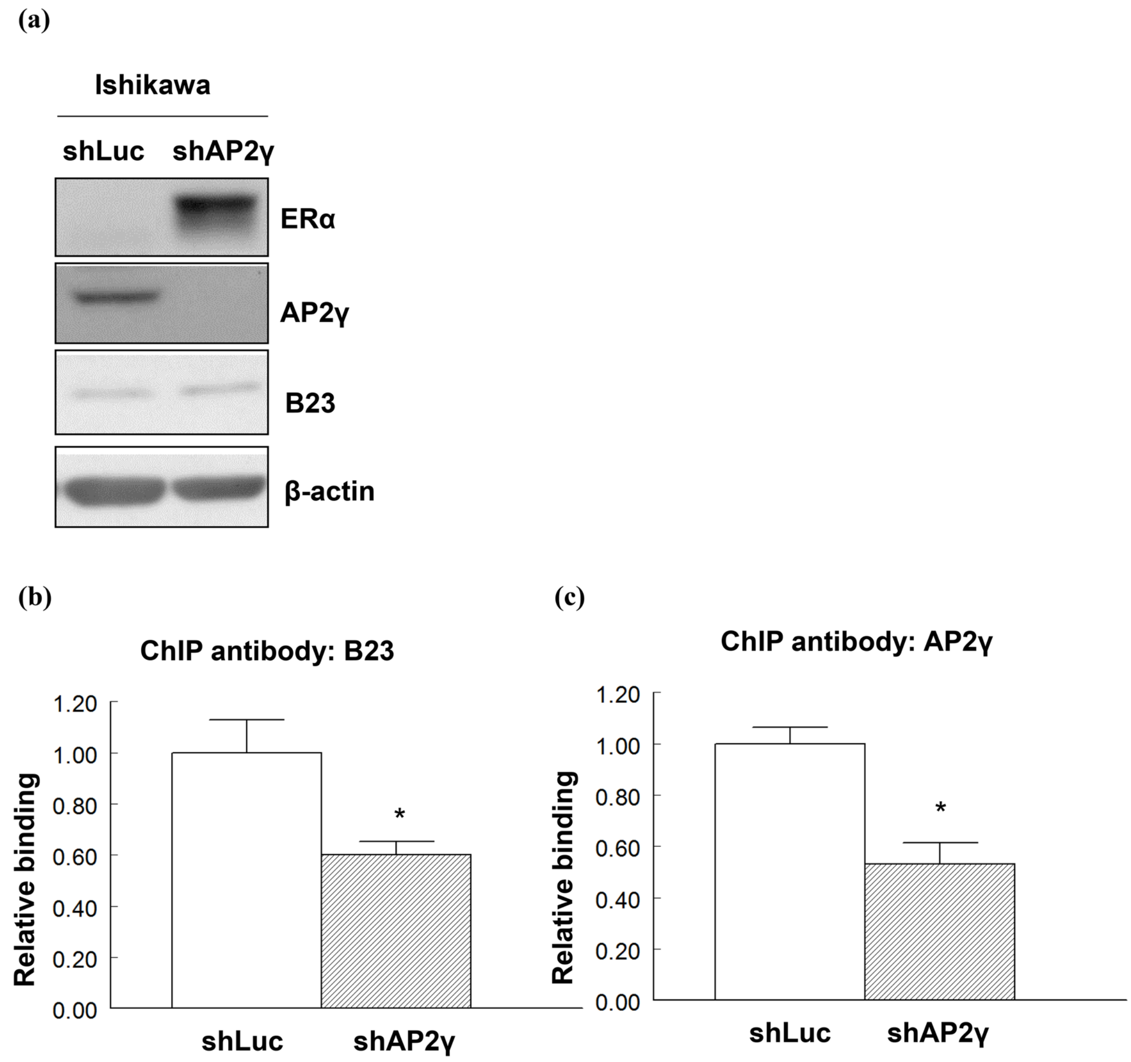

Supplementary Figure S5: The AP2γ recognition site on ERα promoter is required for B23 binding. a. After the isolation of stable knocked-down Ishikawa endometrial cancer cells, equal amounts of protein lysates were separated by SDS-PAGE and subjected to immunoblotting with antibodies raised against ERα, AP2γ, B23, and β-actin. The presence of an equal amount of proteins in each lane was confirmed with β-actin. **b, c.** Chromatin fragments were prepared from Ishikawa shLuc or shAP2γ stable clones for chromatin immunoprecipitation (ChIP) assays. Cell lysates were prepared and immunoprecipitated with control (IgG), anti-B23, and anti-AP2γ antibodies. The immunoprecipitated genomic regions were assayed with real-time qPCR using primers encompassing the AP2γ recognition sequence on the ERα promoter. Data are expressed as means ± standard errors from three independent experiments. * $P < 0.05$ compared with controls.

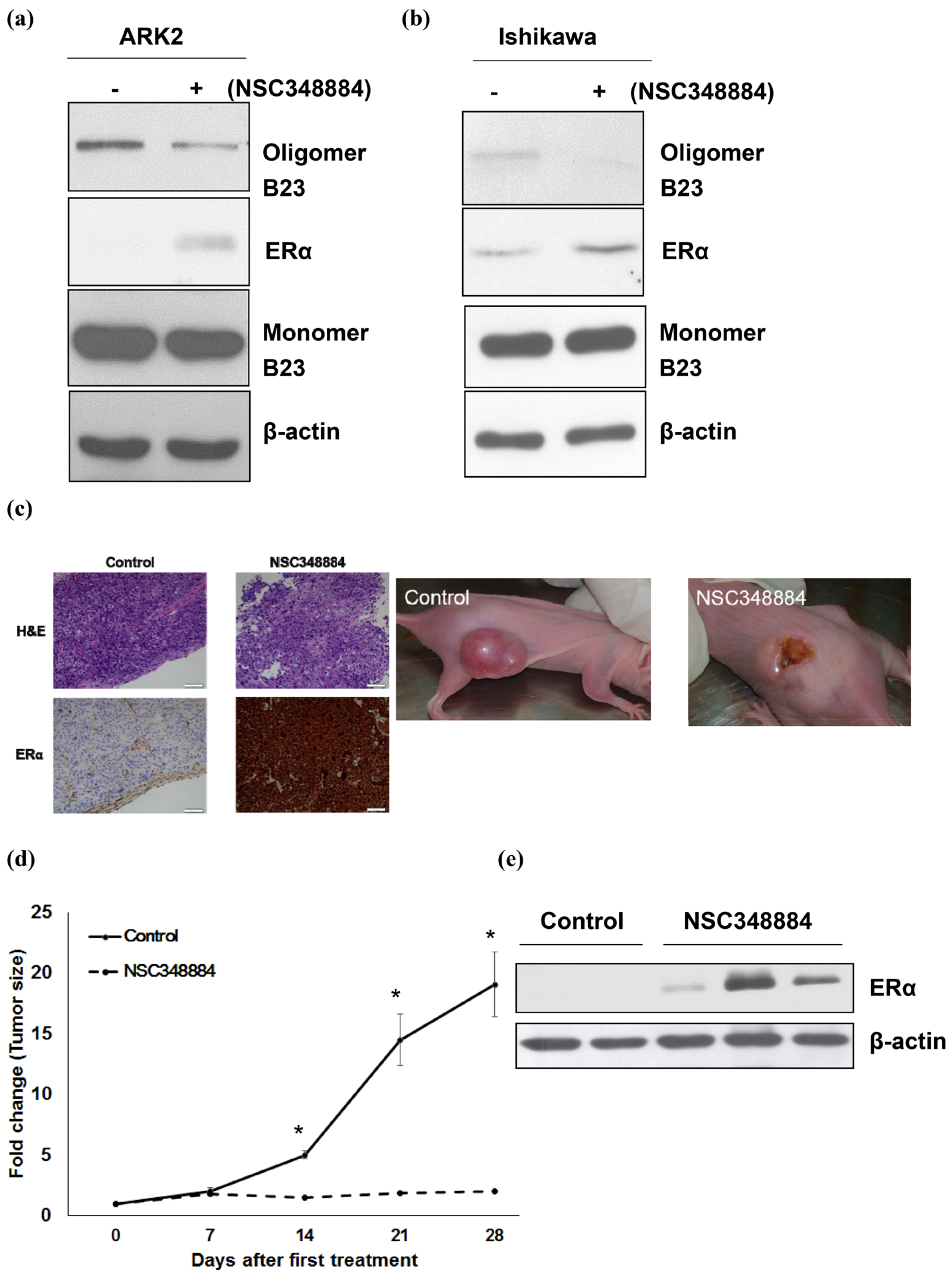

Supplementary Figure S6: NSC348884 specifically disrupts the B23 oligomer and upregulates ERα expression. a, b. ARK2 cells (left panel) and Ishikawa cells (right panel) were exposed to 2.5μM and 5μM of NSC348884 for 24 h. Subsequently, equal amounts of whole-cell extracts were immunoblotted with the reported antibodies. The presence of an equal amount of proteins in each lane was confirmed with β-actin. (c-e) ARK2 endometrial cancer cells were injected subcutaneously into the lateral hind leg of nude mice. Tumors were treated with subcutaneous injections of NSC348884 or a vehicle for 4 weeks. Tumor volumes (cm^3) were measured on a weekly basis. * $P < 0.05$ compared with the NSC348884 group. **c.** Formaldehyde-fixed tumors from mice were sectioned and stained with hematoxylin and eosin. In addition, immunohistochemistry with human ERα antibodies was performed (left panel). Photograph of tumors derived from NSC348884-treated or vehicle-treated tumor-bearing nude mice (right panel). **d.** Tumor growth curve in nude mice. **e.** Tumors treated with NSC348884 or the vehicle were immunoblotted with ERα antibodies. The presence of an equal amount of proteins in each lane was confirmed with β-actin.
